# Oxidative Stress Markers in the Common Bream *Abramis brama* Parasitized with *Ligula intestinalis*

**DOI:** 10.3390/vetsci13040400

**Published:** 2026-04-19

**Authors:** Nadezhda P. Kantserova, Irina V. Sukhovskaya, Albina A. Tsekova, Daria I. Lebedeva, Liudmila A. Lysenko

**Affiliations:** Institute of Biology, Karelian Research Centre of the Russian Academy of Sciences, 185910 Petrozavodsk, Russia; sukhovskaya@inbox.ru (I.V.S.); kochnevaalbina@gmail.com (A.A.T.); daryal78@mail.ru (D.I.L.); l-lysenko@yandex.ru (L.A.L.)

**Keywords:** common bream, *Ligula intestinalis*, plerocercoid, oxidative stress, antioxidant enzymes, vitamins, proteasomes, calpains, protein carbonyls

## Abstract

The present study investigated the effect of *Ligula intestinalis* L. infection on several components of the antioxidant system and on protein oxidation in the host fish, common bream *Abramis brama* L. In ligulosed bream, the hepatopancreatic antioxidant system response included a decrease in catalase (CAT) activity, an increase in glutathione S-transferase (GST) activity, and no change in superoxide dismutase (SOD) activity. The contents of molecular antioxidants in the organs of infected bream were inconsistent; for instance, hepatopancreatic α-tocopherol content was significantly lower, whereas retinol content was significantly higher than in uninfected individuals. In contrast, no significant differences were found in the α-tocopherol or retinol content in the skeletal muscles of infected and uninfected fish. The protein oxidation, estimated via protein carbonyl content, was unaffected by ligulosis, as was the activity of proteasomes. However, the activity of calpain, another protease, was significantly higher in the skeletal muscle of infected fish. Overall, the data reveal moderate and tissue-specific alterations in oxidative stress markers in *A. brama* infected with *L. intestinalis*, suggesting a complex host–parasite interaction that does not result in severe systemic oxidative damage under the studied conditions.

## Simple Summary

Parasitic infections in fish are a common occurrence in natural water bodies, yet their precise physiological effects on the host often remain unclear. This study investigated how infection with the tapeworm *Ligula intestinalis* affects the health of the common bream, a freshwater fish species. The research focused on the fish’s antioxidant defense system, which protects cells from damage, and on protein breakdown processes in the liver and muscles. The findings revealed that the infection triggered moderate changes in the liver’s defensive mechanisms and no significant protein destruction. In the skeletal muscles, increased activity of specific protein-degrading enzymes was detected. The results indicate that while the parasite does affect the host, these changes are limited and do not lead to severe systemic harm. Understanding such host–parasite dynamics is important for assessing the health of fish populations in their natural habitats and contributes to the sustainable management of aquatic ecosystems.

## 1. Introduction

*Ligula intestinalis* (Linnaeus, 1758) Gmelin, 1790 is a common and widely distributed tapeworm whose complex life cycle requires two aquatic intermediate hosts (first copepods, then planktivorous fish) and piscivorous birds as the definitive host [1]. *L. intestinalis* infects more than 170 fish species from different genera, mainly cyprinids [2], and can increase mortality and reduce growth rates in fish populations, with implications for fisheries management [1,2,3].

The plerocercoid, the larval stage of *L. intestinalis*, occupies the abdominal cavity of fish, where it can reside for extended periods and cause ligulosis [4]. The tapeworm *L. intestinalis* is reported to produce multiple effects on fish growth, behavior, reproduction, stress resistance, and the sensitivity of infected individuals to environmental pollution [5,6,7,8,9,10,11]. *L. intestinalis* plerocercoids can significantly affect the behavior of invaded fish by reducing their swimming activity and provoking swimming close to the surface, thus probably facilitating their predation by piscivorous birds, as shown in roach *Rutilus rutilus* Linnaeus, 1758 and *Engraulicypris sardella* Günther, 1868 [6,12]. Upon reaching a large size, plerocercoids lead to increased pressure on internal organs, causing their deformation, necrosis, and dystrophy [5,13].

In fish, *L. intestinalis* plerocercoids also cause reproductive dysfunction. Infected fish have been shown to exhibit suppressed gonad development [7,14] due to reduced production and plasma concentrations of sex steroids, such as 11-ketotestosterone, 17β-estradiol, and progesterone, as well as vitellogenin, as demonstrated in bream [15] and roach [7]. Cytological changes in the pituitary gland and a decrease in gonadotropin synthesis reported in ligulosed roach may also indicate reduced functional activity of the hypothalamic–pituitary–gonadal axis of the host [8].

The effect of *L. intestinalis* plerocercoids on tissue composition and biochemistry in infected fish has been the focus of several studies [16,17,18]. However, the impact of *L. intestinalis* plerocercoid infection on the oxidative status in fish has not yet been fully described [16,17,18]. Substrate oxidation is the basis of cellular energy exchange and a source of free radicals—reactive oxygen species (ROS)—which are involved in physiological processes. When ROS production exceeds the cell’s capacity to eliminate them (due to age, diseases, or unfavorable environmental factors), they can damage cellular components, including organelles, membranes, and macromolecules (lipids, proteins, and nucleic acids). Oxidative stress results from an imbalance between pro-oxidants and antioxidants, leading to an increase in ROS [19]. The accumulation of irreversibly altered (oxidized) macromolecules, including protein carbonyls, is a particular manifestation of oxidative stress [20]. According to current understanding, the antioxidant system includes not only classical enzymes such as superoxide dismutase (SOD), catalase (CAT), glutathione peroxidase (GPx), and glutathione S-transferase (GST), as well as molecular antioxidants like glutathione (GSH), retinol, and tocopherols, but also certain transcriptional factors and proteases of the protein quality control system, such as proteasomes and calpains [21]. To date, only the effects of *L. intestinalis* plerocercoids on the total antioxidant capacity of tissues in common bream [17] and GST activity in roach *R. rutilus* and chub *Squalius cephalus* L., 1758 [18] have been described. It should be noted that antioxidant capacity is an important marker of the physiological state of organisms under unfavorable environmental conditions [22,23], including parasitic infections [24,25].

While previous studies have documented the pathological and reproductive consequences of ligulosis, the specific redox status of the host remains poorly characterized. We hypothesized that chronic infection with *L. intestinalis* would induce a state of oxidative stress in the host, resulting in compensatory modulation of enzymatic (SOD, CAT, GST) and non-enzymatic (vitamins A, E) antioxidants, and culminating in oxidative damage to proteins (protein carbonyls). Furthermore, given the high metabolic demand of plerocercoids and the physical pressure on host organs, we predicted an upregulation of proteolytic systems (calpain and proteasome) to mobilize protein reserves. By analyzing these markers in the hepatopancreas (the primary metabolic organ) and skeletal muscle (the primary protein store), this study aims to delineate the tissue-specific physiological costs of parasitism in a wild cyprinid fish. To our knowledge, this is the first investigation to simultaneously profile the major enzymatic (SOD, CAT, GST), non-enzymatic (vitamins A and E), and proteolytic (calpains and proteasomes) components of the oxidative stress response, alongside quantifying oxidative protein damage (protein carbonyls) in *L.* *intestinalis*–common bream system.

Therefore, the present study aims to quantify some enzymatic and non-enzymatic components of the antioxidant system, as well as protein carbonyl levels, in common bream *Abramis brama* L. 1758 infected with *Ligula intestinalis*.

## 2. Materials and Methods

### 2.1. Sample Collection

*A. brama* specimens were sampled in June 2024 from Kurmoyla Bay (61°53′48.1″ N, 33°05′40.9″ E) of Lake Syamozero, Republic of Karelia, northwestern Russia, using gill nets with mesh sizes ranging from 30 to 60 mm. The water temperature in Kurmoyla Bay was 14 °C. The fish were sacrificed by administering an overdose of clove oil (250 mg/L), individually measured (standard length), and weighed. Thereafter, fish were dissected and examined for *L. intestinalis* plerocercoids according to Dubinina [1]. Infection parameters, including prevalence (percentage of infected host individuals in the sample), intensity of infection (number of parasites per infected host, range), and abundance (mean number of parasites per host individual), were determined. For each ligulosed bream, parasite index (PI, %) was calculated using the formula PI = total weight of *L. intestinalis* plerocercoids/weight of the eviscerated carcass × 100 [26]. Fulton’s condition factor (K) was calculated using the formula: K = 100 × body weight (g)/(body length (cm))^3^. Fish age was determined from scale annuli.

A total of 189 common breams were caught, of which 15 specimens were infected with *L. intestinalis* plerocercoids. The occurrence of *L. intestinalis* and the infection rate of bream in Lake Syamozero were discussed in our previous papers [27,28] and were as follows: prevalence of 8.9% with a ratio of 1.52, intensity of infection ranging from 1 to 3 tapeworms per fish, and abundance of 0.12. For biochemical analyses, the sampled fish were divided into two groups according to infection status (infected vs. uninfected) and standardized by age, sex ratio, and length–weight parameters. In infected fish, the parasite index was similar and ranged from 5.3% to 7.5%. Samples of skeletal muscle and hepatopancreas from 11 infected (7 females, 4 males, age 4+ to 9+) and 11 uninfected (8 females, 3 males, age 4+ to 9+) fish were dissected shortly after euthanasia, immediately frozen in liquid nitrogen, and used for subsequent biochemical analyses. Four infected fish were excluded because they could not be adequately matched with the uninfected group due to differences in size and age. The uninfected fish used as controls were free of *L. intestinalis* and showed no external morphological changes, apparent aberrations in internal organs (particularly skeletal muscle and hepatopancreas), or localized or generalized discoloration, nodules, or cysts.

### 2.2. Reagents and Equipment

Analytical-grade chemical reagents, vitamin standards, protease inhibitors, and substrates were obtained from Sigma-Aldrich (St. Louis, MO, USA). The technical equipment of the Core Facility of the Karelian Research Centre of the Russian Academy of Sciences was used, including a freezing chamber UF 240-86 E (Snijders Scientific, Tilburg, The Netherlands), a TissueLyser LT homogenizer (Qiagen, Hilden, Germany), an Allegra 64R centrifuge (Beckman Coulter, Brea, CA, USA), a CLARIOstar microplate reader (BMG LABTECH, Ortenberg, Germany), and a Millichrom-6 microcolumn liquid chromatograph (Nauchpribor, Oryol, Russia).

### 2.3. Antioxidant Enzyme Assays

After homogenization of frozen hepatopancreas samples (0.1–0.4 g) in 5.0 mM Tris-HCl buffer (pH 7.5) at a ratio of 1:10 (*w*/*v*), the mixture was centrifuged at 45,000× *g* for 1 h at 4 °C. The resulting supernatant was used for enzyme activity assays.

#### 2.3.1. Superoxide Dismutase (SOD) Activity Assay

A modified spectrophotometric method was used to determine SOD activity based on the enzyme’s ability to inhibit adrenaline auto-oxidation in an alkaline environment [29]. Briefly, 10 µL of adrenaline solution (22 mM in 0.1 N HCl) was added to a sample containing an excess of 0.3 M bicarbonate buffer (pH 10.6). The accumulation of adrenochrome, a product of adrenaline oxidation, was recorded spectrophotometrically at 480 nm per minute at 25 °C. SOD activity was expressed in units per mg of protein per minute (U/mg protein/min).

#### 2.3.2. Catalase (CAT) Activity Assay

CAT activity was determined using the method of Beers and Sizer [30]. Enzyme activity was measured by the decomposition of 25 mM H_2_O_2_ in 50 mM phosphate-buffered saline (PBS, pH 7.4) at 25 °C. The optical density of the solution was recorded at 240 nm. Catalase activity was expressed as µmol of H_2_O_2_ decomposed per minute and calculated per mg of soluble protein in the tissue (µmol H_2_O_2_/mg protein/min).

#### 2.3.3. Glutathione S-Transferase (GST) Activity Assay

GST activity was measured using the substrate 1-chloro-2,4-dinitrobenzene (CDNB) to monitor the conjugation of reduced glutathione (GSH) [31]. A reaction mixture (0.225 mL) containing 1 mM CDNB and 1 mM GSH in 0.125 M PBS (pH 6.5) was placed in a microplate well. The reaction was started by adding 0.025 mL of homogenate, and the optical density increase was recorded at 340 nm (25 °C). Activity was expressed as µmol of product per minute per mg of soluble protein (µmol product/mg protein/min).

### 2.4. Vitamin Content Assays

Vitamin concentrations were measured following the protocol described by Skurihin and Dvinskaya [32]. Tissue samples (100 mg) were homogenized in 0.9 mL of 0.25 M sucrose solution (pH 7.4) containing 1 mM EDTA-Na_2_. To precipitate proteins, 0.25 mL of the homogenate was mixed with 0.25 mL of 0.025% butylhydroxytoluene in ethanol. Subsequently, 0.25 mL of 0.0125% butylhydroxytoluene in n-hexane was added, and the mixture was vigorously agitated for 5 min, centrifuged at 3000× *g* for 10 min, and then kept at 4 °C for 40 min. α-Tocopherol and retinol levels were measured in the hexane layer using a Milichrom-6 microcolumn liquid chromatograph equipped with a UV detector set at 292 nm and 324 nm, respectively. Separation was performed on a normal-phase CAC-5-80-4 chromatographic column (Russia) under isocratic conditions at a flow rate of 200 μL/min. The mobile phase consisted of a hexane–isopropanol mixture (98.5:1.5, *v*/*v*). Calibration curves were generated using standard solutions of α-tocopherol and retinol, and vitamin content was quantified by the external standard method, with results expressed as μg per gram of tissue.

### 2.5. Protease Activity Assays

Tissue samples were homogenized in 20 mM Tris-HCl buffer (pH 7.5) containing 150 mM NaCl, 5 mM EDTA, 20 mM dithiothreitol, 1 mM ATP, 5 mM MgCl_2_, and 0.1% Triton X-100, at a ratio of 1:10 (*w*/*v*), and centrifuged at 20,000× *g* for 30 min. The resulting supernatants, containing a pooled fraction of cytoplasmic and organellar proteins, were used for enzyme activity assays. All procedures were performed on ice at 4 °C.

#### 2.5.1. Calpain Activity Assay

To quantify calcium-dependent proteolytic activity, a microplate assay was employed using casein as a substrate [33]. Each reaction mixture (500 µL total volume) contained 0.4% alkali-denatured casein, 20 mM dithiothreitol, 50 mM Tris-HCl (pH 7.5), supplemented with either 5.0 mM Ca^2+^ (as CaCl_2_) for measuring calcium-dependent activity, or 5.0 mM EDTA to chelate calcium for negative controls. After incubation at 28 °C for 30 min, the remaining protein was determined using the Bradford method [34]. One unit of calpain activity (AU) was defined as the amount of enzyme required to increase the absorbance at 595 nm by 0.1 per hour. Specific activity was normalized to the protein concentration in the sample.

#### 2.5.2. Proteasome Activity Assay

A fluorescence assay was employed to measure the chymotrypsin-like peptidase activity of the proteasome [35]. The reaction mixture, which contained 1 mM dithiothreitol, 5 mM MgCl_2_, 1 mM ATP, 30 μM Suc-LLVY-AMC (a synthetic oligopeptide substrate), and 20 mM Tris-HCl (pH 7.5), was incubated with or without 5 μM of the specific inhibitor MG132. After 30 min at 37 °C, proteasome activity was quantified by subtracting the fluorescence intensity of inhibitor-treated samples from that of untreated samples, with excitation at 380 nm and emission detection at 440 nm. The resulting activity was normalized to protein concentration and reported as relative fluorescence fold change (FU).

### 2.6. Protein Carbonyl Assay

The concentration of carbonyl groups in total protein was assessed according to Levine et al. [36]. Tissue was homogenized in 20 mM Tris-HCl buffer (pH 7.5) at a 1:10 (*w*/*v*) ratio and centrifuged at 20,000× *g* for 20 min. A 100 μL aliquot of the supernatant was then mixed with either 500 μL of 0.2% 2,4-dinitrophenylhydrazine (DNPH) in 2 M HCl for experimental samples or 500 μL of 2 M HCl alone for controls. Following a 1 h incubation in the dark at room temperature, 500 μL of 20% trichloroacetic acid was added. The mixture was incubated at 4 °C for 10 min and centrifuged at 10,000× *g* for 10 min. The precipitate was washed twice with ethanol:ethyl acetate (1:1, *v*/*v*), dried, and dissolved in 1 mL of 6 M guanidine hydrochloride by heating to 90 °C. The resulting 2,4-dinitrophenylhydrazones, formed from ketone and aldehyde groups reacting with DNPH, were quantified spectrophotometrically at 370 nm. Absorbance values were converted to concentrations using a molar extinction coefficient of 22 mM^−1^·cm^−1^, and results were expressed as nmol of carbonyls per mg of protein.

### 2.7. Soluble Protein Assay

Soluble protein concentration in the supernatant was assessed spectrophotometrically by measuring peptide bond absorbance at 220 nm at 26 °C [37]; serial dilutions of bovine serum albumin were used as a standard.

### 2.8. Statistical Analysis

All statistical analyses and data visualizations were performed using R [38] within the RStudio integrated development environment [39]. The normality of data distribution was assessed using the Shapiro–Wilk test. A comparative analysis of the indicators was conducted using the Kruskal–Wallis test and Dunn’s post hoc with a correction for multiple comparisons Bonferroni (“rstatix” package).

## 3. Results

### 3.1. Length–Weight Parameters and Fulton’s Condition Factor (K) of A. brama Uninfected and Infected with L. intestinalis

As shown in Table 1, there were no significant differences in length, weight, or K between the two groups of fish.

### 3.2. Biochemical Indices in the Hepatopancreas of A. brama Uninfected and Infected with L. intestinalis

**Figure 1:**
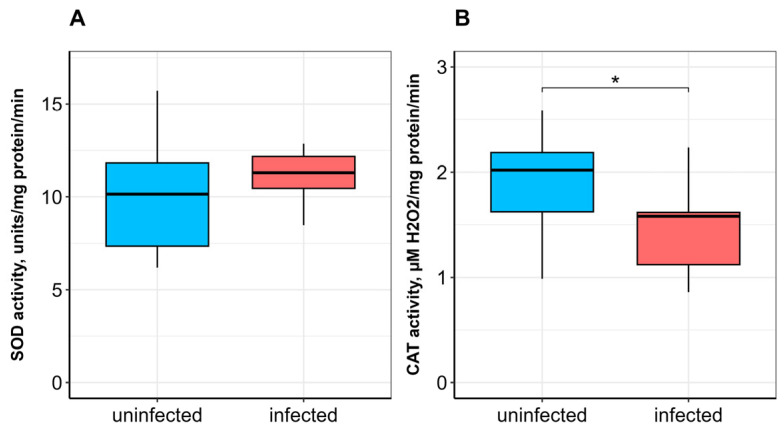
Superoxide dismutase (SOD; (**A**)) and catalase (CAT; (**B**)) activities in the hepatopancreas of *A. brama* uninfected and infected with *L. intestinalis*. Data are presented as median (line within box), interquartile range (box), and min–max values (whiskers). * indicates *p* ≤ 0.05 (Wilcoxon rank-sum test).

**Figure 2:**
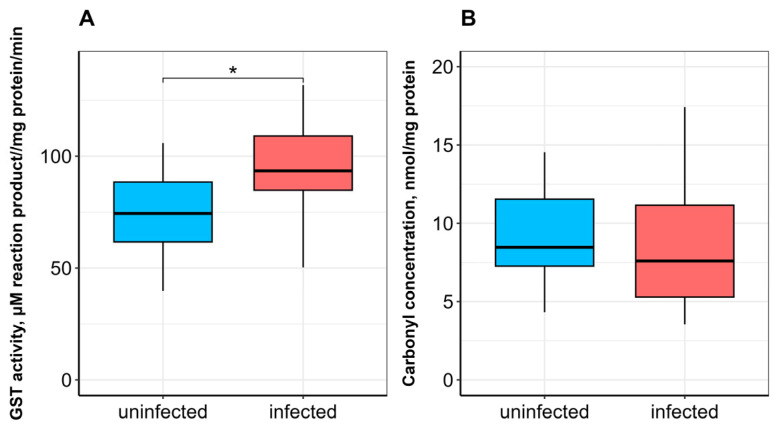
Glutathione S-transferase activity (GST; (**A**)) and protein carbonyl content (**B**) in the hepatopancreas of *A. brama* uninfected and infected with *L. intestinalis*. Data are presented as median (line within box), interquartile range (box), and min–max values (whiskers). * indicates *p* ≤ 0.05 (Wilcoxon rank-sum test).

**Figure 3:**
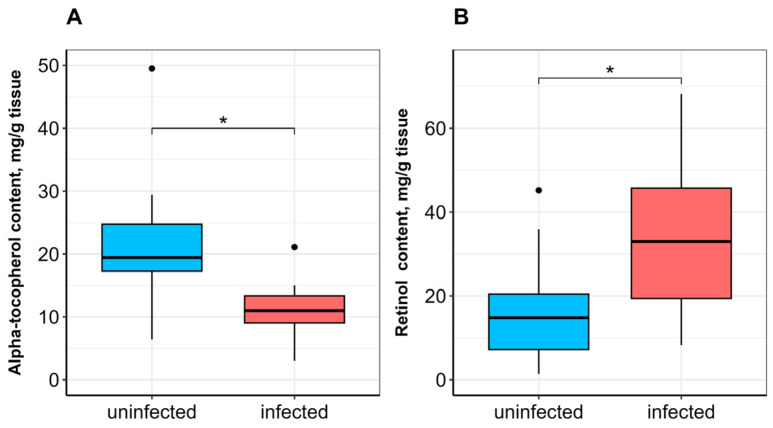
α-Tocopherol (**A**) and retinol (**B**) contents in the hepatopancreas of *A. brama* uninfected and infected with *L. intestinalis*. Data are presented as median (line within box), interquartile range (box), and min–max values (whiskers). * indicates *p* ≤ 0.05 (Wilcoxon rank-sum test).

**Figure 4:**
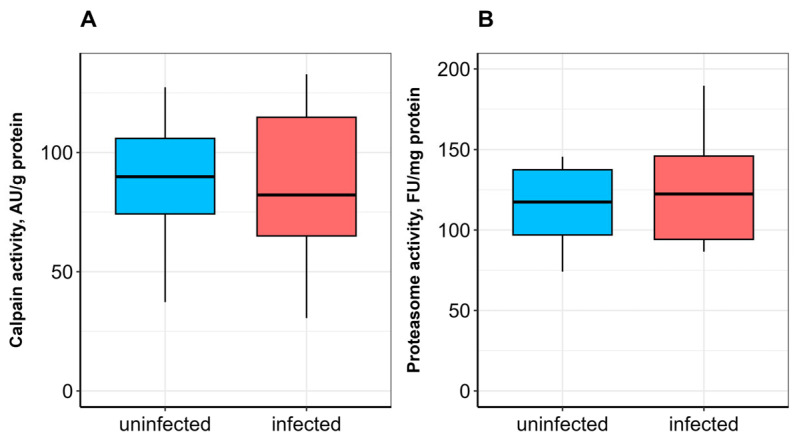
Calpain (**A**) and proteasome (**B**) activities in the hepatopancreas of *A. brama* uninfected and infected with *L. intestinalis*. Data are presented as median (line within box), interquartile range (box), and min–max values (whiskers).

No significant difference in SOD activity was observed between uninfected and infected fish (Figure 1A). CAT activity was significantly lower (*p* ≤ 0.05; Figure 1B), whereas GST activity was significantly higher (*p* ≤ 0.05) in infected fish compared to uninfected ones (Figure 2A). α-Tocopherol content in the hepatopancreas was significantly lower (*p* ≤ 0.05), while retinol content was significantly higher (*p* ≤ 0.05) in infected fish (Figure 3A,B). No significant differences were found in calpain activity, proteasome activity (Figure 4), or protein carbonyl content (Figure 2B) between uninfected and infected fish.

### 3.3. Biochemical Indices in the Skeletal Muscle of A. brama Uninfected and Infected with L. intestinalis

**Figure 5:**
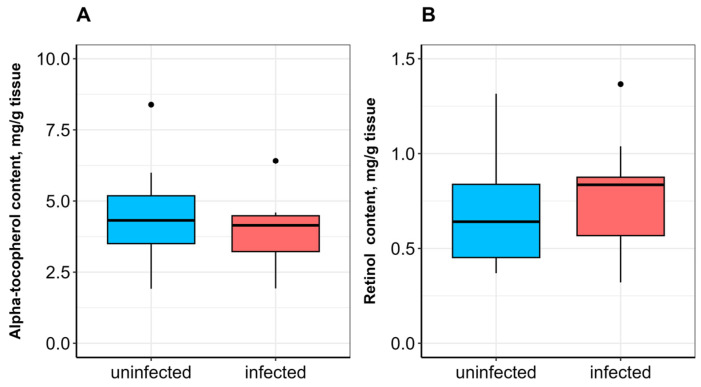
α-Tocopherol (**A**) and retinol (**B**) contents in the skeletal muscle of *A. brama* uninfected and infected with *L. intestinalis*. Data are presented as median (line within box), interquartile range (box), and min–max values (whiskers).

**Figure 6:**
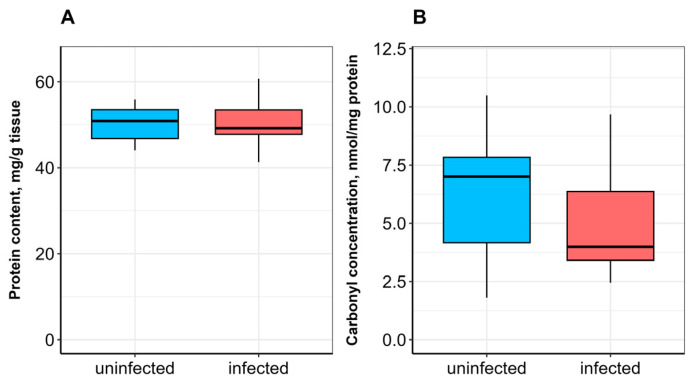
Total (**A**) and carbonylated (**B**) protein contents in the skeletal muscle of *A. brama* uninfected and infected with *L. intestinalis*. Data are presented as median (line within box), interquartile range (box), and min–max values (whiskers).

**Figure 7:**
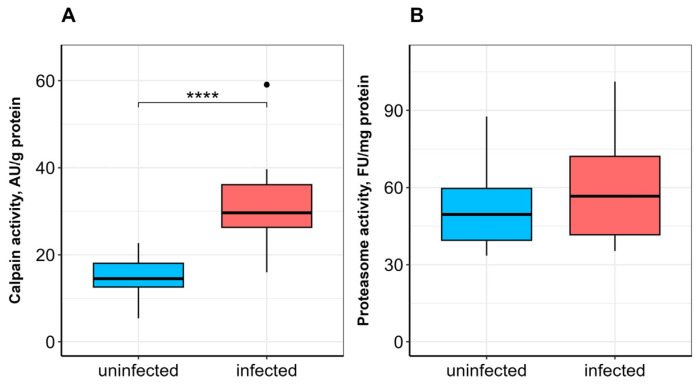
Calpain (**A**) and proteasome (**B**) activities in the skeletal muscle of *A. brama* uninfected and infected with *L. intestinalis*. Data are presented as median (line within box), interquartile range (box), and min–max values (whiskers). **** indicates *p* ≤ 0.0001 (Wilcoxon rank-sum test).

In the skeletal muscle of infected fish compared to uninfected ones, no significant differences were observed in retinol and α-tocopherol contents (Figure 5), soluble protein content (Figure 6A), or protein carbonyl content (Figure 6B). Calpain activity was significantly higher (*p* ≤ 0.0001) in infected bream (Figure 7A), whereas no significant difference in proteasome activity was observed between studied groups (Figure 7B).

## 4. Discussion

In our study, no differences in weight, length, or condition factor (a morphophysiological index reflecting nutritional status, energy metabolism, and nutrient uptake in fish [40]) were observed between ligulosed and non-ligulosed common bream. However, in roach infected with *L. intestinalis*, lower condition factors and even an overall decrease in condition factors, weight, and length have been demonstrated earlier [7,8]. Notably, the aforementioned studies described the effects of *L. intestinalis* infection on young fish (i.e., two-year-olds), which appear to be more susceptible to the influence of the parasite, unlike older age classes. Thus, the similar biometric characteristics of ligulosed and non-ligulosed common bream observed in our study may be explained, at least partially, by the age of the sampled fish (4+ to 9+).

The liver and hepatopancreas, its functional analog in cyprinids, readily respond to unfavorable environmental conditions, as they are involved in metabolism and antioxidant defense through the uptake, biotransformation, and detoxification of pollutants and pathogens [41]. Antioxidant enzymes are commonly used as oxidative stress biomarkers in fish and aquatic invertebrates exposed to adverse environmental conditions [22,23], including parasitic infections [24,25]. It should be noted that oxidative stress associated with parasites can be highly variable, and the response of the host antioxidant system to parasitic infection depends on both the fish species and parasite specificity [25]. Additionally, environmental parameters including pollution, water temperature, dissolved oxygen concentration, and salinity may modulate the antioxidant response in parasitized fish [25,42]. As mentioned above, limited data are available on the effects of *L. intestinalis* on biochemical indices in fish. Therefore, when discussing the results of our study, it is necessary to rely on the available information regarding the impact of parasites from different taxa on biochemical parameters in various host fish species.

SOD and CAT, as rapidly responding antioxidant enzymes, play a key role in the detoxification of superoxide anions and hydrogen peroxide. The dismutation of superoxide by SOD generates hydrogen peroxide (H_2_O_2_), which is subsequently neutralized by CAT [43]. The observed decrease in hepatopancreatic CAT activity, in the absence of increased SOD activity, suggests a specific vulnerability in the H_2_O_2_-scavenging capacity of infected fish. This could lead to an accumulation of H_2_O_2_, which, while not sufficient to cause widespread protein carbonylation (as shown in Figure 2B), may act as a signaling molecule to modulate other pathways, such as the observed increase in GST activity (Figure 2A). This pattern implies that the parasite-induced stress is chronic and low-grade, potentially “priming” the antioxidant system rather than overwhelming it. Van der Oost et al. [22] and Motamedi-Tehrani et al. [44] noted the potential of ROS to impair the antioxidant system in fish. It has been suggested that decreased CAT activity may indicate damage to antioxidant mechanisms caused by excessive hydrogen peroxide production [45]. Our findings (no change in SOD activity and a decrease in CAT activity) are somewhat consistent with data describing a decrease in total antioxidant activity in the hepatopancreas and blood serum of *A. brama* infected with *L. intestinalis* (Rybinsk Reservoir) [17]. Similarly, low SOD activity in the liver, intestine, and skeletal muscle of *Schizothorax plagiostomus* Heckel, 1838 (Cyprinidae) has been associated with infection by acanthocepalan *Pomphorhynchus* sp. [46]. In contrast, elevated SOD and CAT activities in the liver, swim bladder, and intestine of Indian catfish *Wallago attu* Bloch & Schneider, 1801 infected with trematodes *Isoparorchis hypselobagri* Billet, 1898 have been reported [47]. A significant increase in CAT, but not SOD, activity was observed in the liver of Black Sea whiting *Merlangius merlangus euxinus* L., 1758 parasitized by the nematode *Hysterothylacium aduncum* (Rudolphi, 1802) Deardorff & Overstreet, 1981 [48].

GSTs are essential multifunctional enzymes that catalyze the conjugation of reduced glutathione to endogenous and exogenous substances. They also play a role in neutralizing ROS such as O_2_^−^, OH^−^, and H_2_O_2_, thereby protecting the organism from ROS-induced damage [49]. In our study, a significant increase in GST activity was observed in the hepatopancreas of ligulosed bream. The increase in GST activity may represent a compensatory adaptive response, as GSTs not only participate in xenobiotic detoxification but also contribute to the neutralization of ROS and their byproducts, thereby partially compensating for the diminished CAT-mediated H_2_O_2_ scavenging. A similar elevation in GST activity in the liver and intestine has been reported in Indian catfish infected with *Isoparorchis hypselobagri* (Billet, 1898) Odher, 1927 compared to uninfected fish [47]. In contrast, significantly lower hepatic GST activity was found in Black Sea whiting infected with *Hysterothylacium aduncum* (Rudolphi, 1802) Deardorff & Overstreet, 1981 [48] and in roach and chub infected with *L. intestinalis* compared to healthy fish [18].

Vitamins A and E are both lipophilic, food-derived antioxidants that govern fish physiology, but they operate via distinct mechanisms. Vitamin A enhances the organism’s resistance to oxidation due to the ability of retinol (its free alcohol form) to quench singlet oxygen and the ability of retinoic acid (a functionally active metabolite) to increase the expression of antioxidant enzymes [50]. α-Tocopherol, the most abundant form of vitamin E, protects lipids, particularly polyunsaturated fatty acids (PUFAs), from oxidation by scavenging peroxyl radicals without interfering with subsequent chain-propagating steps [51]. In *A. brama*, the highest retinol and α-tocopherol levels were found in the hepatopancreas, with significantly lower levels in skeletal muscle; our results are consistent with data on vitamin accumulation in organs of various fish species [52,53]. In the hepatopancreas of infected bream, α-tocopherol content was significantly lower and retinol content significantly higher compared to uninfected fish. The depletion of α-tocopherol, the primary chain-breaking antioxidant in lipid membranes, strongly suggests its active consumption to counteract lipid peroxidation in the parasite-threatened organ [51]. While the significant depletion of α-tocopherol in the hepatopancreas strongly suggests the occurrence of lipid peroxidation, we did not directly measure lipid peroxidation end products such as thiobarbituric acid reactive substances (TBARS) or 4-hydroxynonenal (4-HNE), which limits our ability to confirm oxidative lipid damage. Conversely, the accumulation of retinol may reflect a specific physiological response. Retinoic acid, a metabolite of retinol, is known to upregulate the expression of GST and other phase II detoxification enzymes [50]. Therefore, the elevated retinol levels in infected fish may not serve as a direct antioxidant per se, but rather as a reservoir for signaling molecules that support the observed increase in GST activity. This could represent a coordinated, two-pronged adaptive response: direct radical scavenging (vitamin E) and indirect transcriptional regulation (vitamin A). No other evidence is currently available regarding the impact of parasites on vitamin A and E content in host fish. However, multiple studies describe the antioxidant role of these vitamins in different fish species in relation to nutritional status [50,54,55], particularly dietary vitamin A in starved *Oncorhynchus mykiss* Walbaum, 1792 [56].

In fish, skeletal muscles make up a large portion of the body (approximately 60% of total weight) and have great metabolic value as they accumulate proteins and lipids [57] for use as energetic substrates. Under optimal conditions, anabolic processes prevail over catabolic ones, ensuring muscle growth, whereas during periods of high energy consumption, such as migration, starvation, or spawning, the breakdown of skeletal muscle proteins predominates [58,59]. Parasites can significantly impact overall physiology and organ integrity in fish through bleeding, damage to internal organs, and impairment of nutrient absorption, resulting in protein loss [60]. In parasitized fish, muscle tissue may be destroyed via myofibrillar protein degradation by proteases [61], leading to significant depletion of protein reserves, as shown in white-sea stickleback *Gasterosteus aculeatus* Linnaeus, 1758 infected with visceral nematodes and *Cryptocotyle* spp. trematodes [62], Koshar fish infected with liver, intestinal, and stomach helminths [60], and several freshwater species infected with acanthocephalan *Pomphorhynchus spindletruncatus* Amin, Abdullah & Mhaisen, 2003 [63]. Conversely, infected fish may exhibit an increase in protein content as a compensatory response to the presence of a parasite [64]. Our study revealed no significant differences in muscle protein content in common bream associated with *L. intestinalis* infection. Similarly, no significant differences in total muscle protein content were observed between uninfected and infected *Lethrinus mahsena* Forsskål, 1775 (infected with Trematodes, Cestodes, and Nematodes) [65].

Protein carbonyls are generated via modification of amino acid residues by ROS following oxidative stress and have been established as a biomarker of oxidative damage [43,66]. Protein carbonylation is irreversible, and the resulting carbonylated proteins are targeted for degradation by proteases due to their increased susceptibility to proteolysis. The absence of significant differences in protein carbonyl levels in the muscle and hepatopancreas of bream in our study indicates no or only moderate pro-oxidant effects of parasite infection. Currently, studies specifically linking helminth infection to protein carbonylation in host fish are unavailable. Nevertheless, in farm animals and cattle, a correlation between protein carbonyl levels and the severity of parasitic infections with gastrointestinal nematodes and protozoa has been demonstrated [67]. Increased protein carbonylation has also been reported in rats parasitized with *Taenia crassiceps* Zeder, 1800 [68] and in Mongolian gerbils infected with *Babesia divergens* M’Fadyean & Stockman, 1911 [69].

In addition to the main components of the antioxidant defense system, proteolysis is also involved in combating the consequences of oxidative stress in cells [21]. The ubiquitin–proteasome pathway is a highly conserved mechanism for recognizing and eliminating oxidized proteins in eukaryotes. In a specific manner, myofibrillar proteins can be degraded via the calpain-dependent pathway [70] in response to increased demand for protein substrates [62]. In our study, no significant differences in the chymotrypsin-like peptidase activity of the proteasome, the rate-limiting step in proteasomal protein degradation, were observed in the muscle or hepatopancreas of bream infected with *L. intestinalis*. By contrast, calpain activity in the skeletal muscle of infected fish was significantly higher than in uninfected fish. The significant upregulation of calpain activity in the skeletal muscle of infected fish, without a corresponding increase in proteasome activity or loss of total protein, points to a specific role for this calcium-dependent protease. Calpains are not primarily responsible for bulk protein turnover (a function of the proteasome and autophagy), but rather for targeted cleavage of cytoskeletal and myofibrillar proteins, often as a prerequisite for tissue remodeling or repair [71]. Given that *L. intestinalis* plerocercoids occupy the body cavity and exert physical pressure on the surrounding musculature, the elevated calpain activity may reflect localized myofibrillar damage and cytoskeletal reorganization rather than a systemic response to starvation. This suggests that the parasite’s impact on muscle is mechanical and structural, whereas the impact on the liver is metabolic and redox-sensitive. Although parasite infestation increases protein consumption through proteasome-independent pathways, the lack of significant differences in protein carbonyl accumulation and proteasome activity probably indicates that the parasite’s impact is insufficient to induce free radical generation and macromolecule oxidation in fish tissues.

This study provides a valuable snapshot of oxidative stress markers in a wild population, but several limitations should be acknowledged. Due to its observational nature, causality cannot be inferred; we cannot determine whether the observed biochemical changes result from the infection or from pre-existing host conditions. Moreover, the sample size (*n* = 11 per group) may be insufficient to detect subtle but biologically relevant changes in highly variable parameters such as muscle protein carbonyls. Another consideration is that wild fish may naturally harbor additional pathogens (e.g., other parasites) either concomitantly with *L. intestinalis* or separately. Although our uninfected controls were carefully screened for visible signs of disease and the absence of *L. intestinalis*, and infected individuals were selected based on the absence of apparent co-infections, the potential influence of undetected or subclinical co-infections cannot be completely excluded. This is an inherent limitation of field-based studies that should be addressed in future research by employing molecular screening for a broader range of pathogens. Finally, the single time point (June) does not account for seasonal fluctuations in parasite burden and host physiology. Consequently, future research should integrate longitudinal sampling with histopathological analysis to assess muscle fiber damage, inflammatory infiltrates, and hepatocellular necrosis, as well as measurements of parasite-specific nutrient uptake, to validate the proposed mechanisms.

## 5. Conclusions

This study provides the first description of the enzymatic and non-enzymatic components of the antioxidant system, as well as protein oxidation, in common bream *A. brama* infected with the tapeworm *L. intestinalis*. The findings demonstrate that parasitic infection induces tissue-specific responses in the host: antioxidant defense mechanisms were predominantly modulated in the hepatopancreas, while skeletal muscle showed enhanced proteolytic activity. The hepatopancreatic response was characterized by decreased catalase activity, increased glutathione S-transferase activity, and opposing trends in vitamin content (decreased α-tocopherol, increased retinol), suggesting a coordinated adaptive response to low-grade oxidative stress. In contrast, skeletal muscle exhibited elevated calpain activity without changes in proteasome activity or protein carbonyl content, indicating a structural rather than systemic oxidative response. These results highlight the complexity of host–parasite interactions and contribute to the fundamental understanding of how helminth infections affect fish physiology, providing a basis for assessing the health status of wild fish populations in natural ecosystems.

## Figures and Tables

**Table 1:** Length, weight, and condition factor (K) of *A. brama* uninfected (*n* = 11) and infected with *L. intestinalis* (*n* = 11). Data are presented as median ± interquartile range (IQR).

Fish	Weight, g	Standard Length, cm	K
Uninfected	199.9 ± 82.5	21.1 ± 2.9	2.02 ± 0.1
Infected with *L. intestinalis*	188.2 ± 82.2	20.7 ± 3.2	1.99 ± 0.1

## Data Availability

The original contributions presented in this study are included in the article. Further inquiries can be directed to the corresponding author.

## Abbreviations

The following abbreviations are used in this manuscript:

4-HNE	4-hydroxynonenal
ATP	Adenosine triphosphate
CAT	Catalase
CDNB	1-chloro-2,4-dinitrobenzene
FU	Relative fluorescence unit
GPx	Glutathione peroxidase
GSH	Reduced glutathione
GST	Glutathione-S-transferase
IQR	Interquartile range
PBS	Phosphate-buffered saline
PI	Parasite index
ROS	Reactive oxygen species
SOD	Superoxide dismutase
TBARS	Thiobarbituric acid reactive substances
